# Mosquito Vectors Survey in the AL-Ahsaa District of Eastern Saudi Arabia

**DOI:** 10.1673/031.011.17601

**Published:** 2011-12-31

**Authors:** Ashraf M. Ahmed, Essam A. Shaalan, Mourad A. M. Aboul-Soud, Frédéric Tripet, Abdulaziz A. Al-Khedhairy

**Affiliations:** ^1^ Department of Zoology, Faculty of Science, King Saud University, P.O. Box 2455, Riyadh 11451, Kingdom of Saudi Arabia; ^2^ Biology Department, Faculty of Science, King Faisal University, P.O. Box 380, Al-Ahsaa 31982, Kingdom of Saudi Arabia; ^3^ Centre of Excellence in Biotechnology Research, Faculty of Science, King Saud University, P.O. Box 2455, Riyadh 11451, Kingdom of Saudi Arabia; ^4^ Centre for Applied Entomology and Parasitology, School of Life Sciences, Keele University, Staffordshire ST5 5BG,UK.; ^5^ Zoology Department, Faculty of Science, Minia University, Egypt; ^6^ Zoology Department, Aswan Faculty of Science, South Valley University, Aswan 81528, Egypt; ^7^ Biochemistry Department, Faculty of Agriculture, Cairo University, Giza, Egypt

**Keywords:** *Aedes caspius*, *Anopheles multicolor*, *Culex perexiguus*, *Culex pipiens*, *Culex pusillus*, Mosquito larvae, seasonal abundance

## Abstract

The present study aimed to identify the mosquito vectors distributed throughout AL-Ahsaa district situated in the eastern region of Saudi Arabia. Mosquito larvae were collected seasonally for one year (October 2009 to September 2010) from different breeding sites in seven rural areas utilizing long aquatic nets. Salinity and pH of these breeding sites were also measured seasonally. The survey revealed the presence of five mosquito species, *Aedes caspius* Pallas (Diptera: Culicidae), *Anopheles multicolor* Cambouliu, *Culex perexiguus* Theobald, *Culex pipiens* L., and *Culex pusillus* Macquart, representing three genera; four of them (*Ae. caspius, An. multicolor, Cx. perexiguus,* and *Cx. pipiens*) are important vectors of diseases. *Ae. caspius* is the most common vector followed by *Cx. pipiens* and then *Cx. perexiguus.* Mosquitoes in AL-Ahsaa are prevalent in both winter and spring seasons, rarely encountered in summer, and are found in moderation during the autumn months. These results are compared with results of other regions in the Kingdom of Saudi Arabia.

## Introduction

Mosquitoes are notoriously undesirable arthropods and are well-known vector-borne diseases (e.g. dengue, filaria, malaria and Rift Valley fever). In Saudi Arabia, the most common mosquito-borne diseases include dengue (Fakeeh and Zaki 2001, 2003; Ayyub et al. 2006; Khan et al. 2008), filaria (Hawking 1973), malaria (Warrel 1993; Abdoon and Alsharani 2003), and Rift valley fever (Jupp et al. 2002; Miller et al. 2002; Al-Hazmi et al. 2003; Balkhy and Memish 2003; Madani et al. 2003). Recently, 76 people have died from an outbreak of Rift Valley fever and 408 people had contracted the disease (Ahmad 2000). The outbreak began in the southern coastal province of Jizan and in the Al Quenfadah and Asir regions of Saudi Arabia. It was the first time to report Rift Valley fever outside Africa since the disease was discovered there in 1930. Three filarial cases were reported from Saudi residences in Armed Forces Hospital, Riyadh in 2002 (Haleem et al. 2002). Omar (1996) reported that local *Culex pipiens* mosquitoes might act as a potential vector of introduced Bancroftian filariasis in Saudi Arabia. Dengue virus was isolated for the first time from an adult in Jeddah, Saudi Arabia in 1994, and from February 1994 through December 2002 the total confirmed dengue cases numbered 319 (Fakeeh and Zaki 2003). Although malaria is endemic to southwestern Saudi Arabia, the number of indigenous malaria cases fell from 467 in 2006 to 58 cases in 2009, with a reduction of 88% (WHO 2010).

Literature review showed that 11 mosquito surveys were conducted in the Kingdom of Saudi Arabia from 1981 to 2005 (Table 1). The work of Mattingly and Knight (1956) could be considered a checklist and full description for mosquito species collected from the Kingdom of Saudi Arabia before 1956. The majority of these surveys (7 of 11) were conducted in southwestern region, while one survey each was conducted in the eastern and middle regions, and one survey each in locations situated in the eastern and western regions. The reason for conducting all the recent mosquito surveys in the southwestern region is due to the epidemic of the Rift Valley fever in 2000; this area, in particular the Asir region, is known as malaria-endemic area. In contrast, surveys conducted in the eastern region, specifically in Al-Ahsaa, were not only rare (Büttiker 1981; Wills et al. 1985), but also not thorough. Since those surveys, no reliable scientific work has been published to clarify the prevalence of mosquito species in the AL-Ahsaa district.

The present study was carried out to morphologically identify mosquito species of medical importance and their prevalence in AL-Ahsaa, in the eastern region of the Kingdom of Saudi Arabia, and to assist in the planning and implementation of mosquito vector control measures in this region.

## Materials and Methods

### Study area

**Figure 1.**
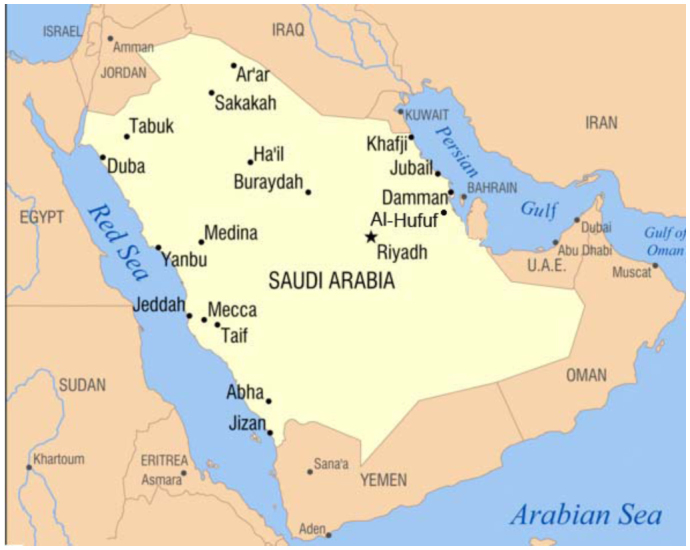
Map of Saudi Arabia showing Al-Hufu, the capital of Al-Ahsaa district. High quality figures are available online.

The present study was conducted in the AL-Ahsaa district, which is situated in the eastern region of the Kingdom of Saudi Arabia (Figure 1). The study of the population dynamics of mosquito larvae was carried out in seven localities (AL-Asfar, AL-Bataliyah, AL-Hufuf, AL-Qurayn, AL'Uqayir, AN-Nuzha, and Ash-Shu'bah) representing urban (AL-Hufuf) and rural areas (all other localities) throughout the AL-Ahsaa district.

### Survey of mosquito larvae

Seasonal larval collections were made from different breeding places from October 2009 to September 2010. Localities were sampled once on 17 October 2009, 17 December 2009, 17 March 2010, and 1 July 2010. Larvae were collected by means of handled larval nets consisting of an iron ring (20 cm in diameter) to which a muslin sleeve (30 cm long) was attached. Samples of three net dips per breeding site were taken from the surface rapidly and gently, by which the number of the larvae were estimated to determine abundance and prevalence of larvae.

The breeding sites were variable, ranging from temporary to permanent. The former included stagnant highly brackish water pools of various sizes, irrigation channels, irrigation and drainage ditches, shores of AL-Asfar lake and AL-Qurayn drainage canal while the latter comprised ground pools constituted by rains, cesspits, and agriculture water catchments. Green algae, short herbs, and upright vegetation were found in several breeding sites. Additionally, some of these sites were rich with rotting organic materials.

**Figure 2.**
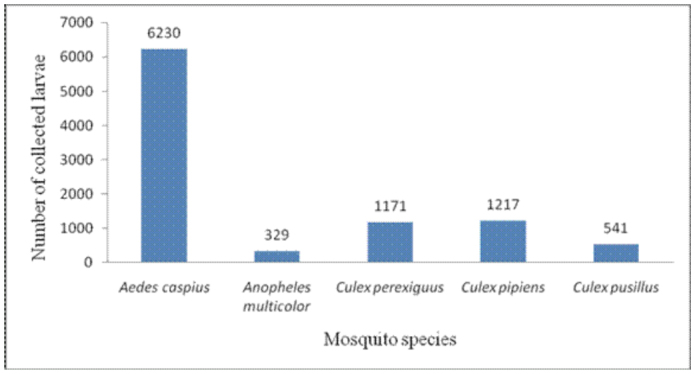
Frequency of mosquito larvae collected from Al-Ahsaa district. High quality figures are available online.

**Figure 3.**
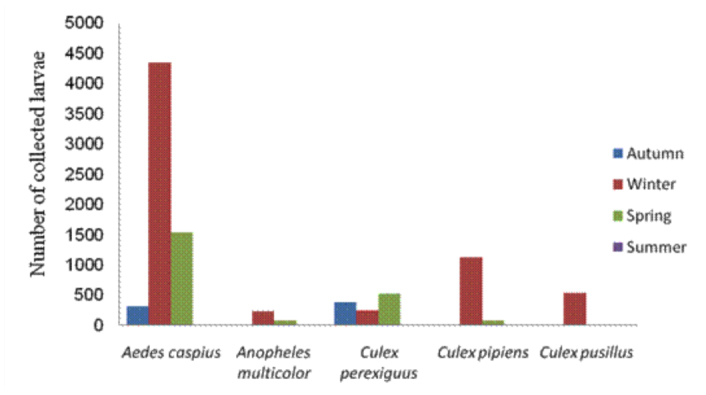
Seasonal abundance of mosquito larvae collected from Al-Ahsaa district. High quality figures are available online.

### Physical characters of breeding sites

Water samples from the breeding sites were transferred to the laboratory for measuring physical characters. Both pH and salinity were measured, and averages of these characters for the breeding sites within each locality can be found in Table 3.

### Larval identification

All larvae were collected with a pipette into ˜ 0.5 L large plastic containers full of clean breeding site water to transport live larvae from the field to the laboratory. Larvae were then killed with 70% alcohol and preserved in glass bottles for identification. Mainly 4^th^ instar larvae were examined and identified according to keys of Abdel-Maleck (1956), Mattingly and Knight (1956), Gad (1963), and Harbach (1985, 1988).

## Results

A total of 9488 mosquito larvae were collected. Results revealed the occurrence of five mosquito species in the study region: one aedine, *Aedes caspius* Pallas (Diptera; Culicidae), one anopheline, *Anopheles multicolor* Cambouliu, and three culicines, Culex *perexiguus* Theobald, *Cx. pipiens* L. and *Cx. pusillus* Macquart.

*Aedes caspius* was the most abundant species in the district, comprising 65.66% (6230 larvae) of the total larval collection (Figure 2). It was encountered in all localities (Table 2), including localities with highly saline water (Table 3). It was collected from permanent and/or temporary highly brackish pools and ditches. It was most prevalent in AN-Nuzha (3480/6230), Ash-Shu'bah (2437/6230), and AL-Qurayn (1169/6230). These localities exhibited the highest salinity levels, ranging from 1.36 to 6.4%, and the highest pH levels, ranging from 7.4 to 8.2 (Table 3). Results in Table 2 and Figure 3 show that the incidence of *Ae. caspius* larvae was higher in winter and spring than other seasons, providing evidence that this species is a cool weather mosquito.

*Culex pipiens* larvae represent 12.83% (1217 larvae) of the total larvae (Figure 2) and were the second most common species collected in this study. This species was detected in all localities except for AL-Asfar, AL-Qurayn, and AN-Nuzha (localities with high levels of water salinity). The larval collections from AL-Hufuf (851/1217) and Ash-Shu'bah (308/1217) gave the maximum population abundance respectively (Table 2), indicating that this species breeds in sites with low and/or moderate salinity (Table 3). Winter season showed the highest incidence of larvae if compared with the other three seasons (Table 2 and Figure 3).

Like *Cx. pipiens, Cx. perexiguus* is also moderately abundant and represents 12.34% (1171 larvae) of the total collection of larvae (Figure 2). It prevails in all localities but with different numbers (Table 2). This species was apparently more abundant in AL-Hufuf (588/1171) and Ash-Shu'bah (292/1171) compared to the other localities (Table 2). These places showed low and/or moderate water salinity, implying that this mosquito species breeds in sites with low and/or moderate salinity (Table 3). The highest peaks of this species were recorded in spring and autumn respectively (Table 2 and Figure 3).

*Culex pusillus* larvae represent 5.7% (541 larvae) of the total encountered larvae (Figure 2). Most of the larvae were collected from AL-Asfar (521 larvae), while very low numbers of larvae (4–11) were recorded from AL'Uqayir , AN-Nuzha, and Ash-Shu'bah (Table 2). This species is restricted to these localities due to occurrence of suitable breeding sites such as a salt lake (AL-Asfar) and brackish pools and ditches (AL'Uqayir, AN-Nuzha, Ash-Shu'bah), indicating that this species is a brackish water species although water salinity was higher in these places. More than 96% (521/541) of *Cx. pusillus* larvae were collected in winter.

Although *An. multicolor* larvae were encountered in most of the localities (AL-Bataliyah, AL-Hufuf, AL-Qurayn, AN-Nuzha, and Ash-Shu'bah), it's abundance was low, comprising only 3.47% (329 larvae) of the total larvae collected (Table 2 and Figure 2). It was collected from locations that were highly variable in their salinity levels, ranging from low to high (Table 3). This species was most abundant in winter (Figure 3).

## Discussion

The present study was conducted to update our knowledge of the prevalent mosquito vectors and their distribution in AL-Ahsaa, located in the eastern region of Saudi Arabia. Surveys revealed the prevalence of five mosquito species *Ae. caspius, An. multicolor Cx. perexiguus, Cx. pipiens,* and *Cx. pusillus.*

These findings add to the previously mentioned surveys (Mattingly and Knight 1956; Büttiker 1981; Wills et al. 1985) conducted in the Al-Ahsaa region. The present survey found species that were not detected in some other studies: Mattingly and Knight (1956) did not detect *Cx. perexiguus,* Wills et al. (1985) did not detect *An.* *multicolor* or *Cx. perexiguus,* and Büttiker (1981) failed to detect any of the mosquito species recorded in our study. Such differences in findings could be due to differences in sampling locations or reliance on taxonomic morphological keys that lead to misidentifications. The latter error lends support for utilizing more reliable techniques such as molecular identification.

In the present study, *Ae. caspius* was the most abundant mosquito (65.66%) followed by *Cx. pipiens* (12.83%), *Cx. perexiguus* (12.34%), *Cx. pusillus* (5.70 %), and *An. multicolor* (3.47%). *Aedes caspius* is widely distributed in different regions of Saudi Arabia such as Riyadh district (Al-Khreji 2005), as well as in the eastern (Mattingly and Knight 1956; Büttiker 1981; Wills et al. 1985) and southwestern regions (Abdullah and Merdan 1995). This particular species was found in all surveyed localities inhabiting highly brackish water bodies (Salinity 1.36–6.4%) and prevailing most of the year, with higher peaks in winter and spring seasons. In agreement with these findings, Wasim (1993) mentioned that larvae of *Ae. caspius* were widely distributed throughout Egyptian salt marshes, salt lake shores, and brackish pools and ditches with peaks of abundance in both February and September. Furthermore, Abdullah and Merdan (1995) mentioned that larvae of this species were encountered in all months and became abundant during March-June in the Asir region of southwestern Saudi Arabia.

Out of seven localities, *Cx. pipiens* (12.83%) was collected from only four. The highest abundances were observed in AL-Hufuf and Ash-Shu'bah, suggesting that it prefers to breed in water bodies with low and/or moderate salinity (Tables 2 and 3). It is also widely distributed in Riyadh district (Al-Khreji 2005), as well as in the eastern (Mattingly and Knight 1956; Wills et al. 1985) and southwestern regions (Abdullah and Merdan 1995; Miller et al. 2002; Abdoon and Ibrahim 2005) of Saudi Arabia. Although it was the second most prevalent species in our study, it was the most common mosquito species in Riyadh district (Al-Khreji 2005), indicating that this species is an urban mosquito. Results of the present survey showed that the highest incidence of this mosquito was recorded in winter. Mohamed et al. (1981) and Kaschef et al. (1982) found that the highest population abundance of *Cx. pipiens* larvae was in the winter season. Contrarily, Abdullah and Merdan (1995) found that abundance of *Cx. pipiens* was relatively high during the period March–November, and Al-Khreji (2005) reported that mosquito abundance decreased during the winter season in Riyadh district.

*Culex perexiguus* was the third most common species in our survey. It was collected from all localities but with different abundances and higher incidence in spring and autumn seasons. Like *Cx. pipiens,* it prevailed in breeding sites with low and/or moderate water salinity (Tables 2 and 3). This is the first time this species has been reported in this region. It was not recorded in AL-Ahsaa or in the eastern region in previous mosquito surveys carried out by Mattingly and Knight (1956), Büttiker (1981), and Wills et al. (1985). It is important to mention that this species was previously reported as *Cx. univittatus* until its status was clarified by Harbach (1985). Jup (1971) showed that *Cx. univittatus* consisted of two morphologically and biologically distinct species, *Cx. univittutus* and *Cx. neavei,* creating a complex. Furthermore, Jup and Harbach (1990) showed that the *Cx. univittutus* complex included three nominal forms: *univittutus, neavei,* and *perexiguus.* Relying completely on taxonomic morphological keys, in particular for this species, is not accurate, and makes clear the importance of utilizing more reliable molecular techniques to avoid misidentifications.

*Culex pusillus* was the fourth most common species. Mattingly and Knight (1956) and Al-Khreji (2005) noted the presence of this species among Saudi Arabian mosquito fauna. Contrarily, Büttiker (1981), Harbach (1985), Wills et al. (1985), Abdullah and Merdan (1995), Miller et al. (2002), and Abdoon and Ibrahim (2005) did not record this species. It was found to prevail on the shores of a salt lake (AL-Asfar) and brackish pools and ditches where water salinity is high, with highest incidence reported in winter. Contrarily, Al-Khreji (2005) mentioned that mosquito abundance decreased in winter in the Riyadh district in Saudi Arabia. Wasim (1993) found that *Cx. pusillus* larvae were widely distributed throughout Egyptian salt lakes, salt marshes, and brackish pools, and were present during summer and autumn with a peak in September.

*Anopheles multicolor* was the only anopheline mosquito collected from Al-Ahsaa. Mattingly and Knight (1956) reported this species in this area, though Büttiker (1981) and Wills et al. (1985) did not. Recent surveys in Riyadh district (Al-Khreji 2005) and the southwestern region (Abdullah and Merdan 1995; Abdoon and Alshahrani 2003; Abdoon and Ibrahim 2005) confirmed that this species is indeed among the anopheline mosquitoes of Saudi Arabia. It is found in locations that vary in their water salinity, and was most abundant in winter, with lower abundance than the other collected species. Abdullah and Merdan (1995) stated that larvae of this species were collected during relatively cold months in Asir region. In contrast, Morsy (1987) reported that *An. multicolor* larvae were common year-round.

In respect to vectorial potential, *Cx. pusillus* is not known to play a role in disease transmission within the region. However, *Cx. pipiens* is known to transmit *Wucheraria bancrofti* the causative agent of Bancroftian filariasisin in this region and other adjacent countries (Hawking 1973; Southgate 1979; Helmy et al. 1981; Harbach 1985), and has been shown to be a vector of the Rift Valley fever virus (Hoogstral 1979; Harbach 1985) and Sindbis virus, which were isolated from mosquitoes caught in the eastern region of Saudi Arabia (Wills et al. 1985). Laboratory studies demonstrated that it is a moderately efficient West Nile virus vector in North America (Turell et al. 2001), and West Nile virus was isolated from this mosquito in Israel (Samina et al. 1986). *Culex perexiguus* is involved in the transmission of pathogens that cause filarial and arboviral disease in humans (Harbach 1985), and West Nile virus and Sindbis virus (Samina et al. 1986), as well as Rift Valley fever virus (Turell et al. 1996), have been isolated from this species. *Aedes caspius* is an efficient vector of Rift Valley fever virus (Turell et al. 1996), and is the vector of Tahyna virus in the Mediterranean region, and harbors some microspridia and the West Nile virus (Milankov et al. 2009). While *An. multicolor* has been incriminated as a malaria vector under experimental conditions (Farid 1981), it is regarded as a secondary malaria vector in some localities of Saudi Arabia (Abdoon and Alshahrani 2003).

In summary, out of five mosquito species existing in Al-Ahsaa district, our study reports *Cx. perexiguus* for the first time in this region. Regarding medical importance, four mosquito species (*Ae. caspius, An. multicolor, Cx. perexiguus,* and *Cx. pipiens*) have been reported as vectors of human borne diseases, while *Cx. pusillus* has no known medical importance. Future research will focus on the molecular identification method for accurately identifying the mosquitoes vectors in Al-Ahsaa district. This may help in designing an accurate disruption map for these vectors, and thus help in the implementation of effective mosquito control measures.

**Table 1.**
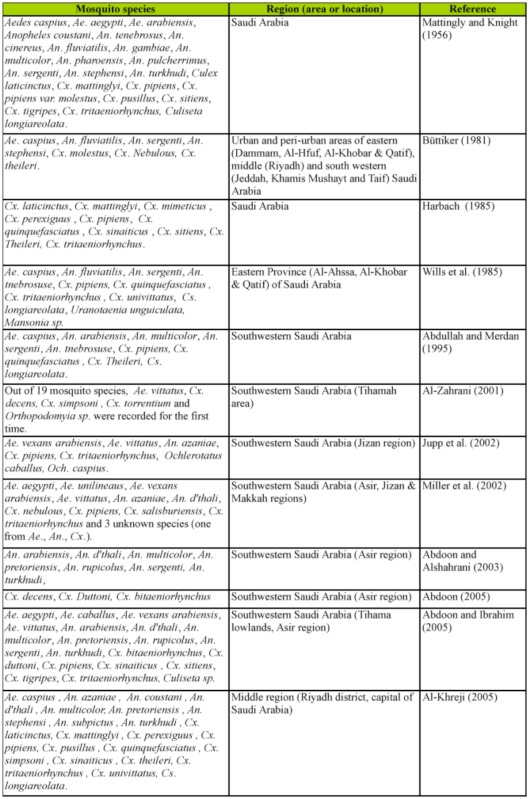
Distribution of mosquito species in Saudi Arabia.

**Table 2.**
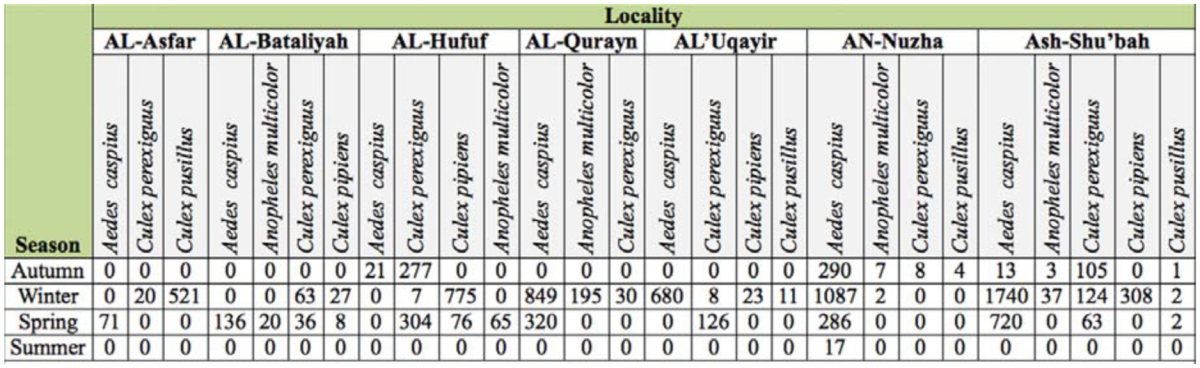
Seasonal frequency of mosquitoes in Saudi Arabia.

**Table 3.**
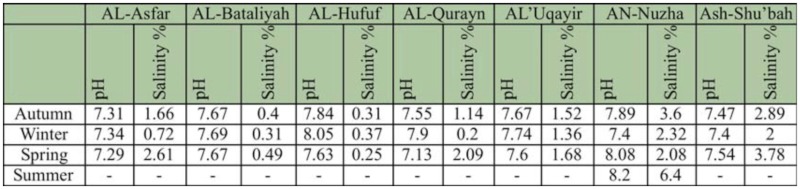
Physical characteristics of breeding sites.
